# Long-Term Weight-Loss in Gastric Bypass Patients Carrying Melanocortin 4 Receptor Variants

**DOI:** 10.1371/journal.pone.0093629

**Published:** 2014-04-04

**Authors:** Bryn S. Moore, Uyenlinh L. Mirshahi, Evan A. Yost, Ann N. Stepanchick, Michael D. Bedrin, Amanda M. Styer, Kathryn K. Jackson, Christopher D. Still, Gerda E. Breitwieser, Glenn S. Gerhard, David J. Carey, Tooraj Mirshahi

**Affiliations:** 1 Weis Center for Research, Geisinger Clinic, Danville, Pennsylvania, United States of America; 2 Geisinger Obesity Institute, Geisinger Clinic, Danville, Pennsylvania, United States of America; University of Santiago de Compostela School of Medicine - CIMUS, Spain

## Abstract

**Background:**

The melanocortin 4 receptor (MC4R) critically regulates feeding and satiety. Rare variants in *MC4R* are predominantly found in obese individuals. Though some rare variants in *MC4R* discovered in patients have defects in localization, ligand binding and signaling to cAMP, many have no recognized defects.

**Subjects/Methods:**

In our cohort of 1433 obese subjects that underwent Roux-en-Y Gastric Bypass (RYGB) surgery, we found fifteen variants of *MC4R*. We matched rare variant carriers to patients with the *MC4R* reference alleles for gender, age, starting BMI and T2D to determine the variant effect on weight-loss post-RYGB. *In vitro*, we determined expression of mutant receptors by ELISA and western blot, and cAMP production by microscopy.

**Results:**

While carrying a rare *MC4R* allele is associated with obesity, carriers of rare variants exhibited comparable weight-loss after RYGB to non-carriers. However, subjects carrying three of these variants, *V95I*, *I137T* or *L250Q*, lost less weight after surgery. *In vitro*, the R305Q mutation caused a defect in cell surface expression while only the I137T and C326R mutations showed impaired cAMP signaling. Despite these apparent differences, there was no correlation between *in vitro* signaling and pre- or post-surgery clinical phenotype.

**Conclusions:**

These data suggest that subtle differences in receptor signaling conferred by rare *MC4R* variants combined with additional factors predispose carriers to obesity. In the absence of complete *MC4R* deficiency, these differences can be overcome by the powerful weight-reducing effects of bariatric surgery. In a complex disorder such as obesity, genetic variants that cause subtle defects that have cumulative effects can be overcome after appropriate clinical intervention.

## Introduction

Obesity is a worldwide epidemic that contributes to comorbidities such as diabetes and heart disease [1]. Severe obesity unresponsive to medication and dieting can be effectively treated with bariatric surgery. Roux-en Y gastric bypass (RYGB), vertical sleeve gastrectomy and gastric banding are the most common bariatric surgeries [2]. Importantly, type 2 diabetes (T2D) often remits following RYGB, before significant weight-loss occurs [3], [4]. RYGB improves blood glucose levels more rapidly and completely than caloric restricted weight-loss or other common bariatric procedures [2], [5].

Regulation of feeding and satiety, essential for maintaining healthy weight, occurs in the hypothalamus. In the fed state, insulin and leptin stimulate neurons expressing proopiomelanocortin (POMC) to release α-melanocyte stimulating hormone (α-MSH) and β-MSH [6]. α-MSH binds and activates melanocortin 4 receptor (MC4R), resulting in an increase in cAMP, inducing the sensation of satiety. Insulin and leptin also inhibit neurons expressing neuropeptide Y (NPY) and agouti-related protein (AgRP). AgRP is a biased agonist of MC4R that stimulates appetite [7]. The balance between the signals from POMC and AgRP neurons critically regulates feeding behavior and energy homeostasis.

The development of obesity, as well as the degree of weight-loss following RYGB surgery can be greatly impacted by genetic variants [8], [9]. For example, extreme obesity can be due to mutations in genes such as *MC4R* [9]–[12]. Common missense variants in *MC4R*, occurring in both lean and obese people at equal frequencies, have also been described [8], [12]–[14]. Recently, we reported that having the common *MC4R* variant *I251L* leads to better weight-loss and weight maintenance following RYGB [8]. Patients with an *I251L* allele also resolved their T2D more quickly than patients with two copies of the *MC4R* reference allele [15]. Numerous rare *MC4R* variants have also been reported, primarily identified in cohorts of obese individuals [16]–[18]. While, some of these rare variants have deleterious effects on MC4R signaling to cAMP (e.g. D90N) [19], binding of agonist (e.g. R18L) [16], or localization (e.g. P299H) [17], many mutations display no known defects. Tao *et. al.* grouped MC4R mutations into five classes: Class I are null mutations; Class II are mutations that cause localization defects; Class III are mutations that cause binding defects; Class IV are mutations that cause cAMP signaling defects; and Class V variants have no known defects [20], [21]. Since many *MC4R* variants have unknown defects, the analysis of each mutation to determine the *in vitro* signaling defects and their possible correlation with obesity phenotypes is necessary.

We report fifteen *MC4R* variants identified in a cohort of 1433 obese patients who underwent RYGB surgery. Of the fifteen variants, two (*V103I* and *I251L*) are common alleles occurring at equal frequencies in both lean and obese populations [8]. The thirteen other *MC4R* variants are rare; twelve were previously reported and one, *G34A*, is novel. Three of the rare variants were associated with poor weight-loss post-RYGB surgery (*V95I*, *I137T* and *L250Q*), one exhibited reduced cell surface expression (R305Q) and two had reduced cAMP signaling (I137T and C326R). Despite the apparent differences in biochemical characteristics, no correlation was found between *in vitro* signaling and pre- or post-surgery metabolic phenotypes. Together these data point to the complex involvement of *MC4R* in obesity, metabolism and weight-loss.

## Materials and Methods

### Study population, clinical variables and sequencing of MC4R

All procedures and patient information were collected under a protocol approved by the Geisinger IRB. All subjects gave written informed consent for this project. Genomic DNA was isolated from blood collected from patients. The sequence for *MC4R* was determined for each sample and matched to clinical data obtained in a de-identified manner through a data broker. A cohort of 1433 patients (79.9% female, median age 46 years (range 18–72 years)) who underwent primary Roux-en Y gastric bypass (RYGB) surgery has been previously described [8]. Subjects were categorized as non-diabetic (HbA1c≤6.0% and no diabetes medications) or diabetic (HbA1c≥6.0% or taking one of four diabetes medications: biguanides, sulfonylureas, insulin, or insulin sensitizing agents). Baseline homeostatic model assessment for insulin resistance (HOMA_IR_) was calculated as HOMA_IR_ = (fasting plasma insulin×fasting plasma glucose)/22.5. DNA extraction from blood samples and *MC4R* gene sequencing was performed as described [8]. We genotyped 451 age and gender matched lean subjects in parallel (68.5% female, median age 52 years (range 25–66 years). RYGB patients were followed 12 months prior to surgery and up to 84 months post-surgery. Blood pressure and pre-surgery weight are presented as three month averages. The time to maximum weight-loss and maximum weight-loss for patients with the *MC4R* reference allele who are non-diabetic, T2D or T2D taking insulin were calculated by plotting the mean body mass index (BMI) and fitted with Hill plot curve as described [8]. Subjects with rare variants were matched with non-carrier patients by Body Mass Index (BMI) (defined as kg/m^2^) (±1), age- (±5 years), gender-, T2D status-, and whether they were taking insulin. Carrier and non-carrier patients' BMIs are plotted during the period 12 months prior to- and up to 84- months after RYGB. Three patients (one with *L250Q*, one with *R305Q* and one with *V253I* variant) could not be matched within these parameters, so the BMI range was extended to ±2–3 and/or the age range was extended to ±8 years.

### MC4R constructs

Individual mutations were made with the Quickchange site-directed mutagenesis kit (Stratagene, Santa Clara, CA, USA) in a 3x HA tagged MC4R in pcDNA3. The 3x HAMC4R was subcloned into pEF6V5:eGFP-CAAX-2A-mCherry (Addgene plasmid 26901) to create the HAMC4R2aGFP construct. A bungarotoxin binding site (MWRYYESSLEPYPD) [22] was added to the N terminus of MC4R by PCR amplification and subcloned into pcDNA3.1. All constructs were confirmed by sequencing of the full length clone.

### Cell Culture

HEK293 cells (ATCC, Manassas, VA, USA) were cultured in MEM with 10% FBS at 37°C and 5% CO_2_. For transient transfections, cells were transfected with plasmids described above by Fugene (Roche, Indianapolis, IN, USA) and used two days post-transfection.

### Cell Surface Receptor Imaging

Bungarotoxin Binding Site (BBS) tagged MC4R constructs (BBS-MC4R) were transfected into HEK 293 cells on poly-L-lysine coated glass bottom Fluorodishes (WPI, Sarasota, FL, USA). Cells were rinsed twice with Imaging Low K containing (in mmol/L): 25 HEPES, 114 NaCl, 2.2 KCl, 2 CaCl_2_, 2 MgCl_2_, 22 NaHCO_3_, 1.1 NaH_2_PO_4_, 2 glucose, pH 7.4, and labeled with 10 µg/mL Bungarotoxin (BTX) conjugated to Texas Red for 10–15 min at 4°C and rinsed again in Low K solution. Live cells were imaged on an inverted Olympus Spinning Disc confocal microscope with IPLab (Beckton-Dickinson, San Jose, CA USA) image acquisition software and processed with ImageJ.

### ELISA

Approximately 10,000 HA tagged construct transfected cells were added to wells of a poly-L-lysine coated 96 well plate. The following day, cells were washed with PBS and fixed with either methanol (for total expression) or 4% paraformaldehyde (for surface expression). Cells were then blocked with 1% milk and incubated in peroxidase conjugated anti-HA antibody. The plate was washed with TBS-T three times and then incubated with 100 µL 3,3′,5,5′-Tetramethylbenzidine Liquid Substrate (Sigma, St. Louis, MO, USA) for 30 minutes. 100 µL of 1 mol/L sulfuric acid was added to each well to stop the reaction. Absorbance was then read at 450 nm on a Spectramax 250 plate reader. The absorbance from untransfected cells was first subtracted and then the cell surface labeled signal was plotted as a percentage of total signal (calculated as the non-permeabilized signal divided by the permeabilized signal ×100) and then normalized to wild-type HA-MC4R expression for that experiment. Significant differences from wild-type were determined using one way ANOVA with Dunnet's post-hoc.

### HAMC4R2aGFP Western

HEK293 cells were transiently transfected with HAMC4R2aGFP or mutant HAMC4R2aGFP, where HA-MC4R and GFP are separated by the 18 amino acid 2a peptide sequence from the foot and mouth disease virus [23], [24]. HAMC4R2aGFP is transcribed and translated as one gene product. After translation, 2a self-cleaves and separates HA-MC4R and GFP into two proteins [23], [24]. Cells expressing HAMC4R2aGFP or mutants were lysed two days post-transfection in lysis buffer (25 mmol/L HEPES, 5 mmol/L MgCl2, 5 mmol/L EDTA and 1%Triton) with protease (cOmplete mini) and phosphatase (PhosSTOP) inhibitors (both from Roche). Protein levels were determined by a BCA assay (Pierce). The lysates were loaded for equal amounts of GFP protein. Lysates were loaded on a 12% Bis/Tris gel and transferred to a nitrocellulose membrane. After blocking with milk and probing with anti-HA (Roche) or anti-GFP (NeuroMAb, Davis, CA, USA) the membrane was developed using SuperSignal West Pico Chemiluminescent substrate using the Fujifilm LAS-4000 (Pierce, Rockford, IL, USA). Intensities of the HA and GFP bands were quantitated and HA intensity was normalized to the intensity of the corresponding GFP band and normalized to the wild-type ratio for that day (n≥3).

### cAMP Microscopy Assay

Cells were transfected with BBS-MC4R constructs and Exchange Protein directly Activated by cAMP (EPAC2)-camps sensor [25], a gift from Drs. Nikolaev and Lohse. Cells were labeled with BTX-Texas Red to identify receptor expressing cells. Images were collected for CFP and Fluorescence Resonance Energy Transfer (FRET) between CFP and YFP. The FRET signal was normalized to the CFP signal for each cell. Baseline cAMP levels were established and then cells were exposed to 100 nmol/L of an MC4R synthetic agonist melanotan II (MTII) and then 100 µmol/L forskolin (for maximum cAMP response).

## Results

We sequenced the MC4R gene of 1433 patients who underwent primary RYGB. A total of eighty patients had fifteen distinct *MC4R* variants. Two of these variants, *V103I* and *I251L*, are common and occur in >1% of both lean and obese populations [26]. The frequencies of *V103I* and *I251L* carriers in the RYGB cohort and 451 lean subjects were similar [8]. The other thirteen variants *S4F (11C>T), G34A (101G>C), H76R (227A>G), V95I (283G>A), T112M (335C>T), I137T (410T>C), F202L (606C>A), L207V (619C>G), L250Q (749T>A), V253I (757G>A), S295P (883T>C), R305Q (914G>A), C326R (976T>C)* are considered rare, and occurred in just eighteen patients. Twelve of these variants have been reported previously [11], [13], [14], [18], [21], [27]–[35]. Of the previously reported variants, *S4F*, *H76R*, *V95I*, *I137T*, *L250Q*, and *C326R* [13], [14], [18], [28]–[33] were only found in obese cohorts; whereas *T112M*, *F202L*, *L207V*, *V253I*, *S295P* and *R305Q* [9], [18], [21], [27], [31], [33]–[35] have been reported in both obese and lean populations. The *G34A* variant has not been previously reported. Notably, no rare variants in *MC4R* were found in our lean cohort.

**Figure 1:**
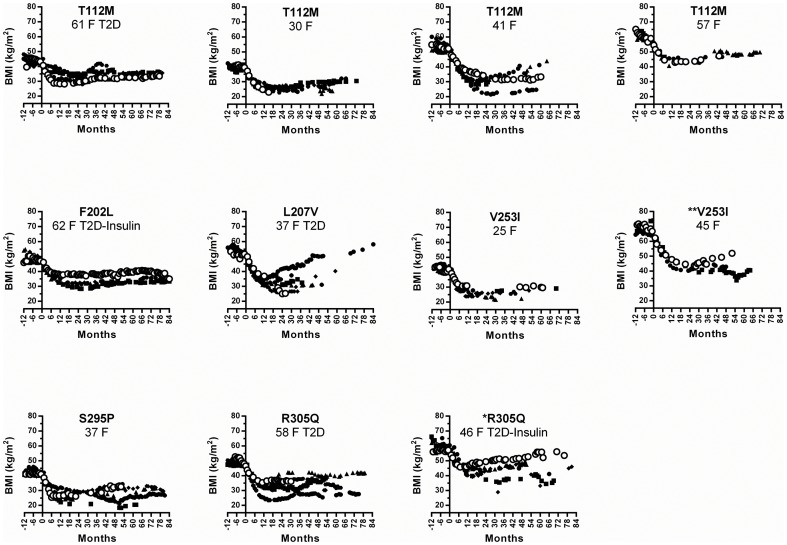
BMIs of patients with rare variants reported in both obese and lean populations. The pre-surgery BMIs of patients with rare variants were matched with non-carrier patients of the same gender, T2D status, insulin medication status and similar age (within 5 years) (black symbols). The variant carrier's age, sex and T2D status is also listed (○). *The starting BMI range for the matches was extended to ±2. **The starting BMI range for the matches was extended to ±3 or the age difference extended to ±8 years.

**Figure 2:**
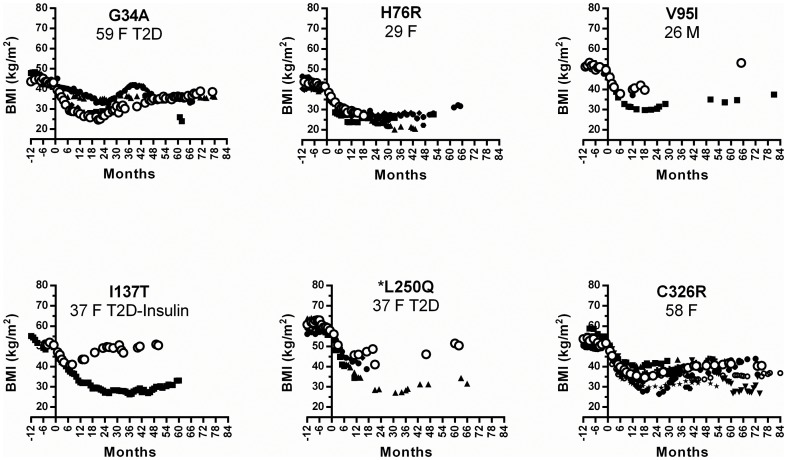
BMIs of patients with rare variants only found in obese populations. The pre-surgery BMIs of patients with rare variants were matched with non-carrier patients of the same gender, T2D status, insulin medication status and similar age (within 5 years) (black symbols). The variant carrier's age, sex and T2D status is also listed (○). *The starting BMI range for the matched patient was extended to ±2.

The *MC4R* variant of each patient and their corresponding body mass index (BMI), T2D status pre- and post- surgery, hemoglobin A1c (HbA1c) (glycated hemoglobin, 3 month blood glucose value), HOMA_IR_ (homeostatic model assessment for insulin resistance) values, and pre-surgery blood pressure are detailed in Table 1. The pre-surgery BMIs of patients with rare variants were not different from patients with the *MC4R* reference allele [15], due to the RYGB selection criteria and the multiple genetic and environmental factors that can contribute to obesity. Of the 18 rare variant carriers, 8 were assessed for binge eating and none were diagnosed. Resting energy expenditure (REE) data was collected for 55% of all RYGB patients, among these the REE of rare variant carriers and non-carriers were not significantly different (data not shown). Following RYGB, most patients lose weight rapidly, reach a maximum weight-loss nadir at ∼10 months and regain some weight in the following months [8]. All patients with *MC4R* variants lost weight following RYGB (Table 1). Patients who achieved maximum weight-loss quickly, lost less weight long-term (Table 1; *V95I*, *I137T*, *R305Q*). To assess whether rare alleles of *MC4R* are associated with weight-loss outcomes following RYGB, we matched each patient carrying a rare *MC4R* variant with patients with the reference sequence for gender, age, starting BMI, and T2D status (non-diabetic, T2D, T2D-taking insulin) (Figures 1 and 2). The post-RYGB weight-loss of patients carrying rare *MC4R* variants reported in obese and lean cohorts (*T112M*, *F202L*, *L207V*, *V253I*, *S295P* and *R305Q*) were indistinguishable from matched controls (Figure 1). Similarly, the weight-loss following RYGB of patients with *MC4R* variants *G34A*, *H76R* and *C326R*, found in obese only populations, were similar to matched controls (Figure 2). Insufficient data were available to assess the *S4F* mutant. Patients carrying the *V95I*, *I137T* and *L250Q* variants did not achieve the same weight-loss compared to control patients (Figure 2). Interestingly, these variants have only been reported in obese individuals [13], [14], [31]. We also assessed overall weight-loss by patients using either loss of initial BMI (%BMI) or percent excess body weight loss (%EBWL), where excess body weight is defined as weight above BMI of 25. Using either one of these criteria, patients carrying *V95I*, *I137T* and *L250Q* did not achieve the same weight loss as others. However, the overall odds of achieving the same RYGB results, %BMI loss or %EBWL as defined above, were not different for patients carrying rare variants or non-carriers.

**Table 1:** Clinical variables for Roux-en Y gastric bypass patients with rare *MC4R* variants.

DNA change	Protein Change	Gender (F/M)	Pre-surgery BMI (mean BMI ± SEM)	Time to lowest BMI (months)	Lowest Recorded BMI (mean BMI ± SEM)	Last Recorded BMI (mean BMI ± SEM)/month	Pre-surgery HbA1c (%)	Pre-surgery HOMA_IR_ (Arbitrary Units)	Pre-surgery Blood Pressure SBP/DBP (mmHg)	Pre-surgery Type 2 Diabetes Status	On Insulin Prior to Surgery	Type 2 Diabetes Remission @ 6 Months Post Surgery (remitted/total)
	**MC4R**	599/125	47.9±0.3	9.9	32.9±0.1	35.0±0.3/49	5.8±0.0	5.4±0.3	135±0.5/79±0.3	No	-	-
	**MC4R**	327/94	48.4±0.4	9.3	33.2±0.1	35.3±0.4/50	6.6±0.1	8.5±0.5	136±0.7/78±0.4	Yes	No	372/396*
	**MC4R**	128/56	49.0±0.6	9.3	34.5±0.2	35.7±0.5/50	8.2±0.1	13.5±1.7	140±1/75±0.7	Yes	Yes	73/175*
**11C>T**	**S4F^¶^**	F	59.5±1.3	No data	No data	No data	15.4	19.5	143±11/78±12	Yes	No	No data
**101G>C**	**G34A**	F	42.9±0.2	21	25	38/77	no data	4.6	137±10/79±3	Yes	No	Yes
**227A>G**	**H76R**	F	41.4±0.1	18	27	27/18	5.9	2.1	132±7/87±0.6	No		
**283G>A**	**V95I**	M	49.7	6	38	53/65	5.5	7.2	154/97	No		
**335C>T**	**T112M**	F	43.7±0.2	14	28	33/78	6.9	4.2	108±3/65±3	Yes	No	Yes
**335C>T**	**T112M**	F	39.9±0.2	14	23	23/15	5.4	2.7	149±7/81±7	No		
**335C>T**	**T112M**	F	52.0±0.2	53	31	33/60	5.6	0.8	141±3/81±7	No		
**335C>T**	**T112M**	F	57.8±1.3	15	43	47/44	5.9	1.9	142±7/68±5	No		
**410T>C**	**I137T**	F	51.3±0.7	6	41	50/50	6.3	3.1	130±10/82±2	Yes	Yes	Yes
**606C>A**	**F202L**	F	47.4±0.2	84	35	35/84	7.5	18.2	117±10/71±12	Yes	Yes	No
**619C>G**	**L207V**	F	51.3±0.2	23	25	25/26	5.8	20.1	121±6/72±4	Yes	No	Yes
**749T>A**	**L250Q**	F	58.9±0.5	21	41	50/62	5.2	3.1	136±17/84±7	Yes	No	Yes
**757G>A**	**V253I**	F	40.8±0.6	23	27	30/61	no data	5.7	151±3/76±4	No		
**757G>A**	**V253I**	F	67.6±0.8	28	43	52/52	6	11.6	116±3/67±4	No		
**883T>C**	**S295P**	F	41.3±0.3	6	25	33/52	5.4	4.5	143±9/87±11	No		
**914G>A**	**R305Q**	F	48.9±0.6	11	35	36/30	9.7	14.5	146± 12/77±7	Yes	No	No
**914G>A**	**R305Q**	F	56.8±0.3	7	46	54/76	6.3	4.3	109±10/65±7	Yes	Yes	Yes
**926T>C**	**C326R**	F	51.3±0.4	18	35	40/75	5.1	2	127±8/78±10	No		

Non-carrier patients are separated into non-diabetics, type 2 diabetics (T2D) and T2Ds taking insulin. Pre-surgery BMIs and blood pressure are reported as three month averages. No data were collected after surgery for the S4F (denoted ¶).

*The total number of patients differ in the six month remission data because those patients that did not have enough follow-up data collected were excluded from analysis (T2D-25 patients, T2D insulin dependent-9 patients).

**Figure 3:**
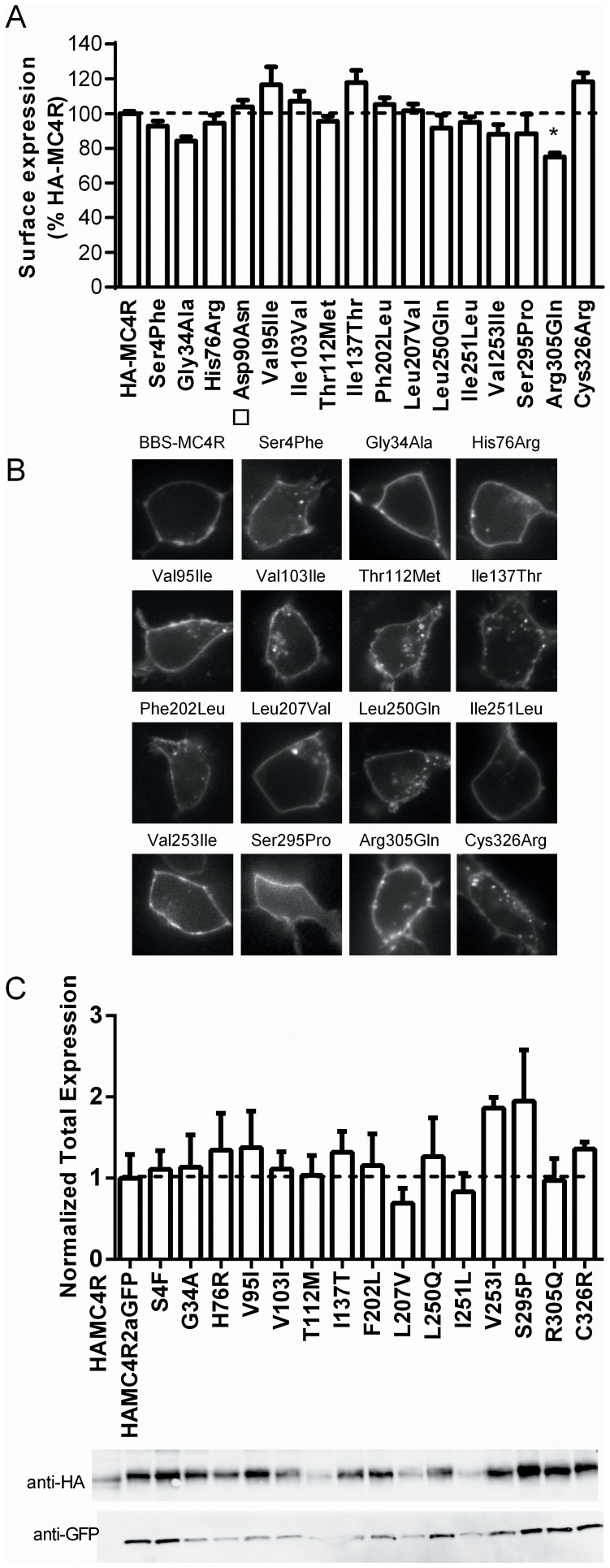
Expression of MC4R mutants. A) ELISA of HEK293 cells expressing wild-type MC4R or mutations. The cell surface expression was normalized to total expression for each mutant and then to wild-type receptor for that batch (* denotes p<0.05 compared to wild-type by a one way ANOVA with Dunnet's post-hoc test). □ MC4R mutant D90N was not found in our cohort. B) Cell surface localization of HEK-293 cells expressing mutant or wild-type (BBS-MC4R) constructs labeled with Bungarotoxin-Texas Red. C) Normalized GFP loading reveals no differences in HA-MC4R mutant lifespan. HA-MC4R expression was normalized to GFP expression and plotted as a percentage of wild-type for each blot (n≥3).

To better understand the potential molecular mechanisms associated with the MC4R mutations, we measured expression and canonical signaling *in vitro*. For each of the fifteen *MC4R* variants in the RYGB cohort, we analyzed the cell surface localization by ELISA and imaging of cell surface labeled receptors. Approximately 40% of total MC4R was located at cell surface and all mutants showed similar percent cell surface expression. Only the R305Q mutation displayed a small but significant decrease in cell surface expression compared to the wild-type receptor (Figure 3A). We examined the cell surface expression of these variants by imaging a bungarotoxin binding sequence (BBS) tagged-MC4R. Labeling of BBS-MC4R and mutants with membrane impermeant bungarotoxin-Texas Red [22] showed that all mutant receptors were effectively expressed on the cell surface (Figure 3B). Labeled receptors were present in the cytoplasm of cells expressing the mutants or wild-type receptor, indicating some receptor internalization.

In order to determine if there were differences in total expression of the MC4R protein *in vitro*, we assayed expression by western blot analysis. We employed an HA-MC4R2aGFP construct where HA-MC4R2aGFP is transcribed and translated as one gene product, but the 2a peptide self-cleaves co-translationally creating equal numbers of two distinct proteins HA-MC4R and GFP. To account for different transfection efficiencies, we loaded equal amounts of GFP protein and assayed for expression of HA-MC4R. Any differences in MC4R expression must be due to changes in protein lifespan of the mutants, since GFP was initially transcribed and translated at a 1∶1 ratio with MC4R. We found that the expression of the mutant receptors was not different from the wild-type receptor (Figure 3C).

**Figure 4:**
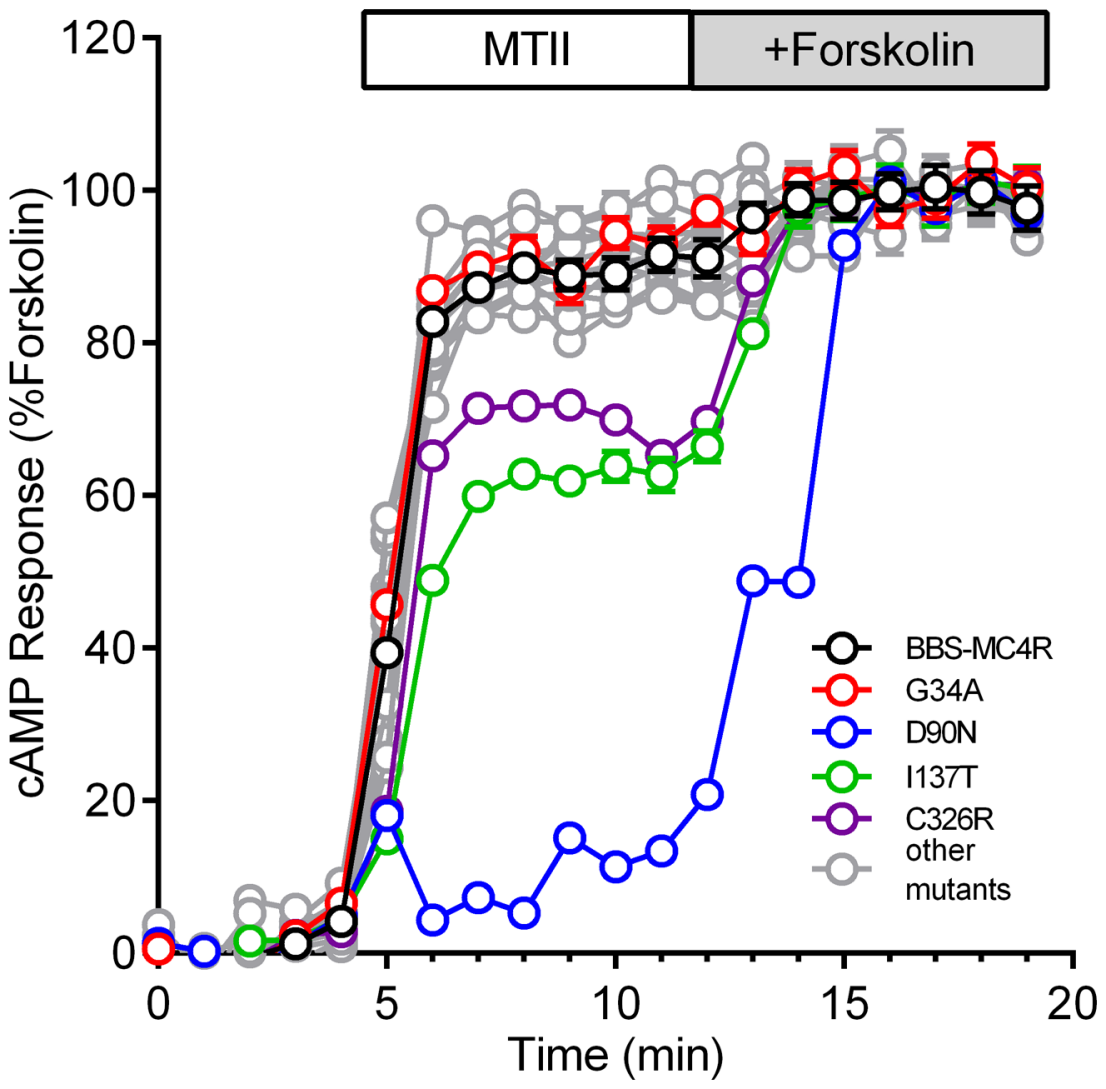
cAMP Assay of MC4R mutants. cAMP production of MC4R mutants after stimulation with 10 nmol/L melanotan II (MTII) and then 100 µmol/L forskolin to activate maximum receptor-independent cAMP response. MTII stimulation was normalized to baseline cAMP production and plotted as a percentage of forskolin in the same cell. Mutants similar to wild-type (Black ○) are designated with the symbol (Gray ○). MC4R novel mutant G34A (Red ○) and those that have different statistically altered cAMP signaling (p<0.01 compared to wild-type by a one way ANOVA with Dunnet's post hoc test) (I137T (Green ○), D90N (Blue ○) and C326R (Purple ○)) are highlighted with different colored symbols. The D90N variant was not found in this cohort, but included as a control.

We next assayed the ability of the MC4R mutants to signal to the cAMP pathway using a FRET based sensor (Epac2-camps) [25]. We expressed BBS-MC4R and mutants, selected cells expressing similar levels of receptor based on the bungarotoxin-Texas Red labeling and determined the cAMP response to the MC4R agonist melanotan II (MTII), normalized to the maximal response induced by forskolin in the same cell (Figure 4). We included the D90N mutation of MC4R as a negative control, because a previous report showed that this mutant had normal cell surface localization and agonist binding but reduced cAMP signaling [19]. The I137T and C326R mutants had a small but significant impairment of cAMP signaling compared to the wild-type receptor (p<0.01), whereas all other mutants responded equally to the MTII stimulus (Figure 4).

The G34A variant has not been reported previously. We measured the MC4R activated cAMP production using an EIA on cells stably expressing wild-type MC4R and G34A and found no difference in α-MSH EC_50_ for cAMP production (Figure S1). The α-MSH induced cAMP production for the remaining mutants have been reported and our data is consistent with the literature for both cell surface and cAMP signaling for the previously reported mutants [13], [27], [28], [31], [33], [36], [37]. The cAMP EIA corroborates the FRET data and validates the imaging assay as a useful single cell measure of cAMP with the ability to determine the maximum cAMP level in the same cells and to select cells expressing similar levels of receptor.

## Discussion

We and others have reported that, in general, carrying an *MC4R* variant is not deterministic for poor outcome after RYGB [15], [38]–[40]. However, in all of those studies the relationship between the cellular signaling for a given mutant and the post-RYGB outcome in the subjects carrying the corresponding variants were not examined. Examining the effects of *MC4R* rare variants in weight-loss after surgery by matching subjects for other variables minimizes confounding factors that cannot be eliminated or addressed in population studies of obesity. We matched *MC4R* variant carriers to non-carriers for the most critical common predictors of weight-loss: gender, age, starting BMI, and diabetic status [41] to ask whether carrying an *MC4R* variant that does or does not affect expression or function can be predictive of post-RYGB phenotype. We find that only 3 patients, carrying *V95I*, *I137T* and *L250Q*, failed to lose comparable weight to matched patients with reference alleles. While these variants have only been reported in obese populations, we did not find a consistent and clear defect in their expression, trafficking and signaling, except for ∼25% reduction in maximal cAMP signaling by I137T. Surprisingly, variants that displayed alterations in expression (R305Q) or signaling (C326R) had no significant phenotypic consequences after RYGB. We conclude that carrying a rare variant of *MC4R*, while associated with obesity, does not affect weight-loss after gastric bypass surgery. This is consistent with reports of weight-loss in rodents after RYGB where only complete loss of MC4R function had a significant effect on weight-loss [15], [39].

These findings compel us to propose a dual role of MC4R in obesity and clinical outcomes after gastric bypass:

### Post-RYGB outcomes

Post-RYGB weight-loss provides a short-term measurement in a more controlled setting, due to the physical influence of the procedure and the clinical follow-up conducted post-surgery. Our data show that modest deleterious effects of *MC4R* variants may not play a significant role in weight-loss following RYGB, similar to the effect seen in heterozygous *Mc4r* mice [15]. In contrast, *Mc4r* null mice fail to lose weight after RYGB [15]. Together the mouse and human data show that one functional copy of MC4R is necessary and sufficient to lose weight after RYGB. Nevertheless, a significant proportion of our patients do not respond to RYGB. Our findings suggest that the response of those patients to weight-loss is independent of their MC4R activity and may arise from other genetic and non-genetic factors. However, carriers of the *I251L* allele, which *in vitro* has increased basal activity [36], more effectively lose weight and resolve their T2D [8], [15]. Additionally, selective receptor re-expression in neurons of *Mc4r* knock-out mice normalized weight-loss and insulin resistance in mice after gastric bypass surgery [15].

### Obesity

A wealth of data exist that show association between carrying rare, presumably deleterious, *MC4R* variants and obesity. Our data corroborate these findings since we only found rare variant carriers in the obese population [8]. We speculate that even modestly altered MC4R activity can influence obesity due to a long-term effect that can be exacerbated by environmental factors such as food choice and/or variants in other obesity related genes. These effects are observed in rodents where heterozygous *Mc4r* mice develop obesity on high fat diet or even regular chow; however, on reduced fat diet these mice do not gain significant weight compared to their wild-type littermates [42]. Reduced food intake in *MC4R* rare variant carriers can result in significant weight-loss, but an increased effort is needed to stay on a weight-loss regimen [43]. While we do not have feeding behavior data for our patients, all patients are placed on a calorie restricted diet for 6–12 months in order to achieve some weight loss prior to RYGB. In our cohort during the 6 month pre-surgery calorie restriction period, carriers of any rare *MC4R* allele lost 6.6±0.9% of their initial weight, while non-carriers lost 6.1±0.2% of their initial weight. Therefore, allele carriers can lose similar weight during calorie restriction suggesting again that in the absence of environmental factors *MC4R* variants do not affect the ability to lose weight. While *MC4R* rare variants are highly associated with obesity, external factors contribute significantly to the obese phenotype. At the cellular level no consistent change in MC4R signaling can account for the association of rare variants with obesity. For example, only two of the rare mutations, I137T and C326R, had defects in cAMP signaling, while the other mutations signaled as well as wild-type MC4R. The defects of the I137T and C326R would only account for ∼25% reduction in function of one copy of *MC4R*. This small defect may contribute to the obesity of those carrying these variants; however, only the *I137T* carrier failed to lose similar weight compared to a matched subject post-RYGB. While there may be a common unifying mechanism for all the obesity associated *MC4R* variants, none has been reported to date.

Together these data highlight the complex nature of MC4R signaling and how it affects not only obesity and metabolism, but also weight-loss. Altered MC4R function does not rule out the effect of other factors that may impact both obesity and RYGB outcomes. We suggest that increasing MC4R function will have a positive outcome on weight-loss after caloric restriction or RYGB, as well as improved T2D remission after RYGB. While several MC4R agonists have failed in clinical trials, mainly due to unrelated side effects, they do hold significant promise for weight-loss, which may depend on concomitant modification in feeding behavior.

## Supporting Information

Figure S1 cAMP dose-response of MC4R and variant G34A. cAMP dose-response in HEK cells stably expressing HA-MC4R (○ solid black lines) and the mutant G34A (□ dashed line) from three independent immunoassays. The α-MSH EC_50_ value for WT-MC4R is 41.1 nM and for G34A is 43.0 nM which were not statistically different (ANOVA with Dunnet's post-hoc test).
(TIF)

Methods S1 **Supplemental Methods.**
(DOCX)
