# Supplementary figures and images for: Long-Term Weight-Loss in Gastric Bypass Patients Carrying Melanocortin 4 Receptor Variants
Source: PLoS One. 2014 Apr 4;9(4):e93629. doi: 10.1371/journal.pone.0093629 (PMC3976318; doi:10.1371/journal.pone.0093629)

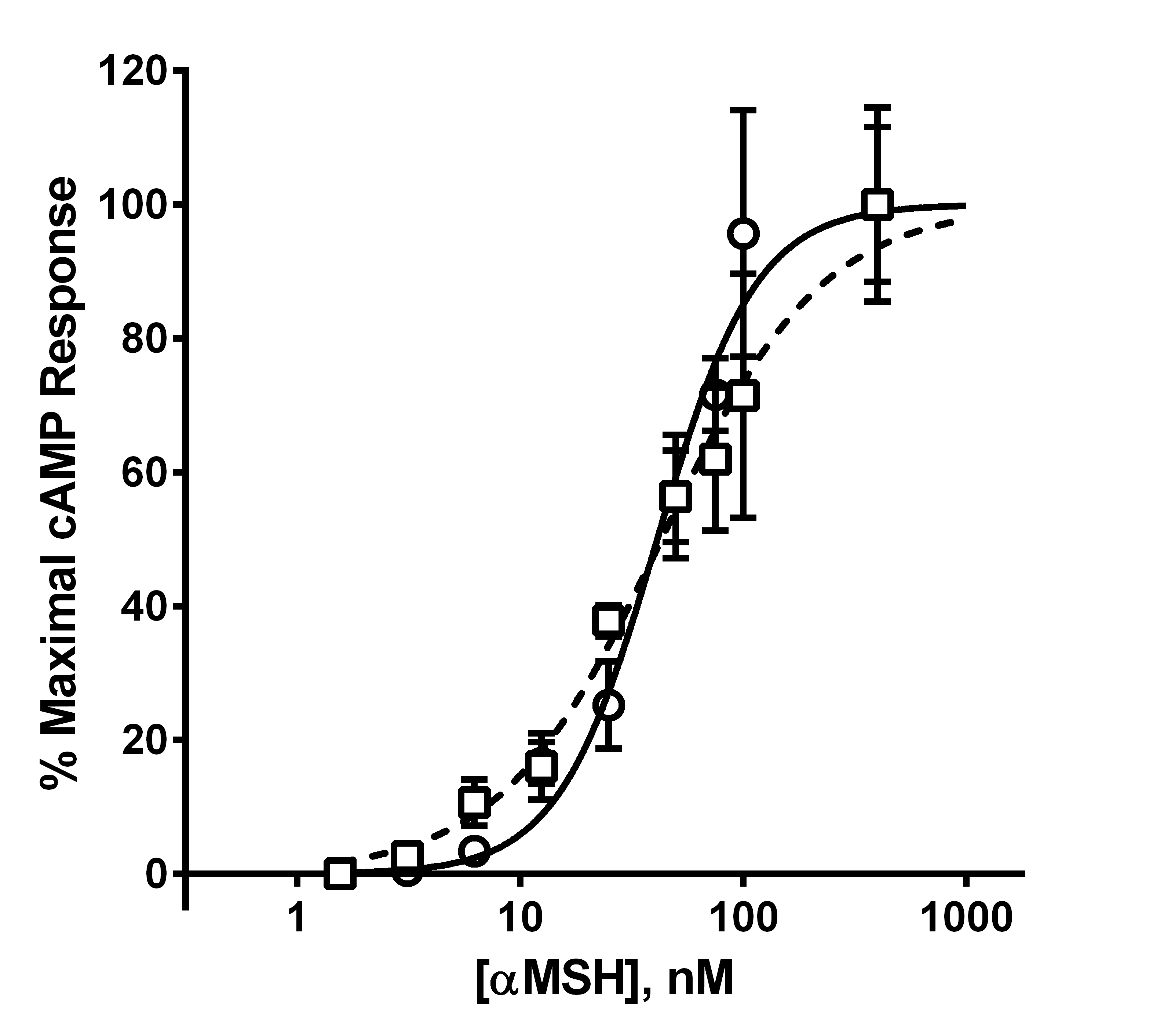

Supplement: Figure S1 — cAMP dose-response of MC4R and variant G34A. cAMP dose-response in HEK cells stably expressing HA-MC4R (○ solid black lines) and the mutant G34A (□ dashed line) from three independent immunoassays. The α-MSH EC50 value for WT-MC4R is 41.1 nM and for G34A is 43.0 nM which were not statistically different (ANOVA with Dunnet's post-hoc test). (TIF) [file pone.0093629.s001.tif]
