# Supplementary material for: Long-Term Weight-Loss in Gastric Bypass Patients Carrying Melanocortin 4 Receptor Variants
Source: PLoS One. 2014 Apr 4;9(4):e93629. doi: 10.1371/journal.pone.0093629 (PMC3976318; doi:10.1371/journal.pone.0093629)
Supplement: Methods S1 — Supplemental Methods. (DOCX) [file pone.0093629.s002.docx]

**Supplemental Methods**

*cAMP* *Enzyme-linked Immune Assay (EIA) Assay*. Cells stably transfected with HA-MC4R, G34A or L207V were stimulated with 100 µM forskolin or 0.1-300 nM α-MSH in MEM+10 %FBS for 10 min. The media was removed and the cells were then lysed with 0.1M HCl and 1% Triton and briefly centrifuged. The supernatant was then used as per the instructions in a cAMP EIA assay (Enzo, Plymouth Meeting, PA, USA). cAMP amount was determined by interpolation of values from the standard curve and normalized to forskolin response for each construct. The normalized cAMP values were used to construct the dose response curves for each MC4R mutant, from which the EC_50_ was calculated using Prism (GraphPad Software, Inc., San Diego, CA, USA). Statistical differences between the logEC_50_ of the curves were compared via global fitting and F test, followed by Bonferroni’s multiple comparisons post hoc.
